# Effects of Specific Training Programs on Punch Performance

**DOI:** 10.3390/sports14050194

**Published:** 2026-05-08

**Authors:** Manuel Pinto, João Crisóstomo, Christopher Kirk, Javier Abián-Vicén, Luís Monteiro

**Affiliations:** 1 CIDEFES, Faculdade de Educação Física e Desporto, Lusófona University, 1749-024 Lisbon, Portugal; jcpcrisostomo@gmail.com (J.C.); luis.monteiro@ulusofona.pt (L.M.); 2 Sport and Physical Activity Research Centre, Sheffield Hallam University, Collegiate Crescent, Sheffield S10 2BP, UK; c.kirk@shu.ac.uk; 3 Performance and Sport Rehabilitation Laboratory, Faculty of Sport Sciences, University of Castilla-La Mancha, Avda. Carlos III s/n, 45071 Toledo, Spain; javier.abian@uclm.es; 4 ICPOL Research Center, Higher Institute of Police Sciences and Internal Security, 1300-352 Lisbon, Portugal

**Keywords:** combat sport, functional strength development, exercise prescription for power training, punch impact power

## Abstract

Punch impact power is crucial for boxing performance and varies with punch biomechanics. Straight punches rely primarily on linear force production, whereas Hook punches depend more on rotational and lateral force generation; however, the effectiveness of strength and conditioning (S&C) interventions remains insufficiently explored. This study investigated the effects of targeted S&C programs on Straight and Hook punch impact power in trained boxers compared with regular boxing training. Thirty-one boxers completed an eight-week intervention and were allocated to three groups: a Linear-Oriented Training Group (LOTG), a Rotational-Oriented Training Group (ROTG), or a Control Group (CG). Punch impact power (Jab, Cross, Lead Hook, and Rear Hook) was assessed using PowerKube at baseline and post-intervention. One-repetition maximum bench press (1 RM BP), countermovement jump (CMJ), and handgrip strength (HS) were also evaluated. Data were analyzed using mixed-design repeated-measures ANOVA and one-way ANOVA on post–pre change scores (Δ). A significant main effect of time was observed for all punch types (*p* < 0.001), with significant group × time interactions for the Cross, Lead Hook, and Rear Hook (*p* < 0.05). The ROTG showed the greatest improvements, particularly in Hook punches. Targeted S&C interventions, particularly rotational training, improved punching impact power and neuromuscular performance.

## 1. Introduction

Boxing performance depends on the effectiveness and power generated through different punching techniques [1]. Punches are typically classified into Straight punches (executed in the sagittal plane with a linear trajectory), Hooks (performed in the transverse plane with a lateral trajectory), and Uppercuts (executed in the sagittal plane with an upward trajectory) [1]. Among these, Straight punches and Hooks are considered particularly decisive due to their central role in offensive and defensive strategies [2]. The primary objective in boxing is to land effective strikes while avoiding counterattacks, thereby controlling the bout [3]. Consequently, punch mechanics and impact have received considerable scientific attention [4,5], with punch impact power commonly assessed using reliable instruments such as the PowerKube (PK), a portable system equipped with high-precision accelerometers that transmit data to dedicated software for accurate measurement of punching impact power [6].

Beyond technical execution, punch selection is influenced by distance, positioning, and movement relative to the opponent, with body height (BH), armspan (AS), and body mass (BM) playing important roles in punch effectiveness and impact power [1,7,8]. From a biomechanical perspective, Straight punches—particularly the rear-hand Cross—rely on rapid force transfer through the kinetic chain, emphasizing lower-body drive, trunk rotation, and arm extension in the sagittal plane [9,10]. In contrast, Hook punches involve a more circular motion, with force generation largely derived from rotational momentum and transverse-plane force production [11]. Given these mechanical distinctions, it is plausible that targeted strength and conditioning (S&C) programs could enhance performance by addressing specific force demands—linear force production for Straight punches and rotational strength for Hook punches. However, limited research has directly compared the effects of such targeted interventions with standard boxing training.

From a training perspective, programs integrating strength, power, and high-intensity exercises have demonstrated effectiveness in bringing about the physiological adaptations required in combat sports [12]. Performance improvements depend on maximal or repeated high-intensity efforts over short time frames. In this context, plyometric exercises—such as explosive jumps—play a key role in optimizing punching mechanics and efficiency [13]. Moreover, complex training methods, which combine maximal strength exercises with power exercises, have shown positive effects on punch power. For instance, pairing heavy bench press sets (5 reps at 5 RM) with medicine ball throws enhances punching force [14]. In addition, resistance-based methods that apply external load to sport-specific punching movements—using cables, bands, medicine balls, barbells, or dumbbells—are widely implemented in boxing practice and are proposed to promote neuromuscular adaptations aligned with punching biomechanics [15]. Recent evidence synthesized in a review of full-contact combat sports indicates that both specific and nonspecific S&C interventions can enhance striking force through improvements in maximal strength, power output, and core force transmission [16]. Despite this, most boxing training programs continue to follow a traditional model emphasizing repeated technique practice, bag work, and sparring [15]. While such methods contribute to technical and conditioning development, they may not optimally enhance the underlying physical determinants of punch effectiveness. Research suggests that targeted resistance training can significantly improve sport-specific performance, particularly when exercises are biomechanically aligned with competition movement patterns [17]. However, the extent to which this principle applies to boxing remains underexplored.

Taken together, previous research has demonstrated significant associations between muscle performance—particularly strength and power—and punch impact, with correlations reported for one-repetition maximum bench press (1 RM BP), countermovement jump (CMJ), and handgrip strength (HS) [3,18,19,20]. Anthropometric and physical conditioning attributes therefore appear to be key determinants of boxing performance [21]. From a practical perspective, taller athletes may exploit their reach advantage through Straight punches, to keep their opponent out of range and off balance [3]. In this context, these considerations highlight the importance of anthropometric characteristics in boxing performance, with BM being the primary determinant of competitive classification, while body mass index (BMI) may provide complementary information regarding athletes’ anthropometric profiles [22]. However, limited experimental research has examined how S&C programs targeting specific force orientations influence Straight and Hook punch performance. Therefore, this study aimed to investigate the effects of targeted S&C interventions emphasizing linear or rotational force production on Straight and Hook punch impact power, compared with standard boxing training. It was hypothesized that (i) training emphasizing linear force production would result in greater improvements in Straight punch impact power; (ii) training emphasizing rotational and transverse force production would elicit greater improvements in Hook punch impact power; (iii) both targeted S&C interventions would produce greater improvements in punch impact power across all punch types compared with standard boxing training; and (iv) both targeted S&C interventions would produce greater improvements in 1 RM BP, CMJ, and HS compared with standard boxing training. These hypotheses were primarily tested through group × time interaction effects derived from mixed-design repeated-measures ANOVA models.

## 2. Methods

### 2.1. Procedure

This study employed a randomized experimental design to compare the effects of two short-term sport-specific S&C training programs with a regular boxing program. The study protocol was developed following the SPIRIT guidelines [23] and presented in a flowchart according to the CONSORT guidelines [24]. Forty-five boxing practitioners from the same gym were randomly assigned to three groups: the Linear-Oriented Training Group (LOTG, *n* = 15), the Rotational-Oriented Training Group (ROTG, *n* = 15), and the Control Group (CG, *n* = 15).

**Figure 1:**
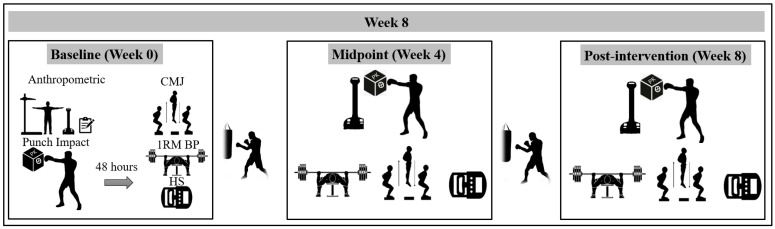
Schematic representation of the study design. CMJ = countermovement jump; HS = handgrip strength; 1 RM BP = 1 repetition maximum bench press.

During the initial laboratory visit, athletes completed the validated 7-day Physical Activity Recall (7-day PAR) to assess their physical activity habits [25], underwent anthropometric measurements, and performed punch impact power tests using the PK device. Forty-eight hours later, participants returned to the laboratory to perform a series of testing procedures, including the CMJ, 1 RM BP, and HS assessments. All testing sessions were conducted between 8:00 and 11:30 a.m. under similar temperature (24–25 °C) and humidity conditions (48–51%) to control for potential diurnal variations in performance. The experimental procedures are detailed in Figure 1.

### 2.2. Strength and Conditioning Interventions

The LOTG and ROTG groups followed a specialized S&C program tailored to enhance specific punching techniques—Straight punches for LOTG and Hooks for ROTG—on Mondays and Fridays. The training programs were designed and supervised by certified S&C coaches and researchers with more than 10 years of experience in combat sports, including boxing, and in S&C planning and programming. All participants (LOTG, ROTG, and CG) followed a standardized regular boxing program focused on technical skills five days per week (Monday to Friday) (Table 1). The training intervention lasted for eight weeks. The training programs for each group are detailed in Table 2 and Table 3, outlining the number of sessions, intensity, and specific exercises of the intervention. External training load was quantified using exercise-specific intensity prescriptions. For resistance exercises performed with barbells, dumbbells, and cable machines (e.g., hexagonal bar deadlift, bench press, and cable row), intensity was prescribed relative to individual strength levels using percentages of 1 RM and adjusted throughout the intervention according to participants’ ability to complete the prescribed repetitions. Specifically, loads were increased when participants were able to perform more repetitions than prescribed and reduced when they were unable to complete the target repetitions. In the ROTG, elastic resistance was standardized by using the same elastic band (20 kg nominal resistance) for all participants across sessions and instructed to perform the concentric phase of each movement as fast as possible under the supervision of the coaching staff. Participants were scheduled to be assessed on three occasions using the PK instrument: at baseline, at the midpoint of the intervention (four weeks from baseline), and at the end of the eight-week training period. However, only participants who completed the intervention and all assessment sessions were included in the final statistical analyses, following a per-protocol approach. Because all participants included in the final analyses completed both baseline and post-intervention assessments, no missing outcome data required imputation procedures. Therefore, an intention-to-treat analysis was not required in the present study. Nevertheless, its methodological importance for minimizing potential attrition bias in randomized trials is acknowledged. Participants included in the final analyses met predefined attendance requirements for the intervention period, corresponding to participation in at least 85% of the scheduled training sessions. Attendance was monitored throughout the intervention by the research staff. Mean adherence rates were 92% (LOTG), 95% (ROTG), 90% (CG).

### 2.3. Participants

**Figure 2:**
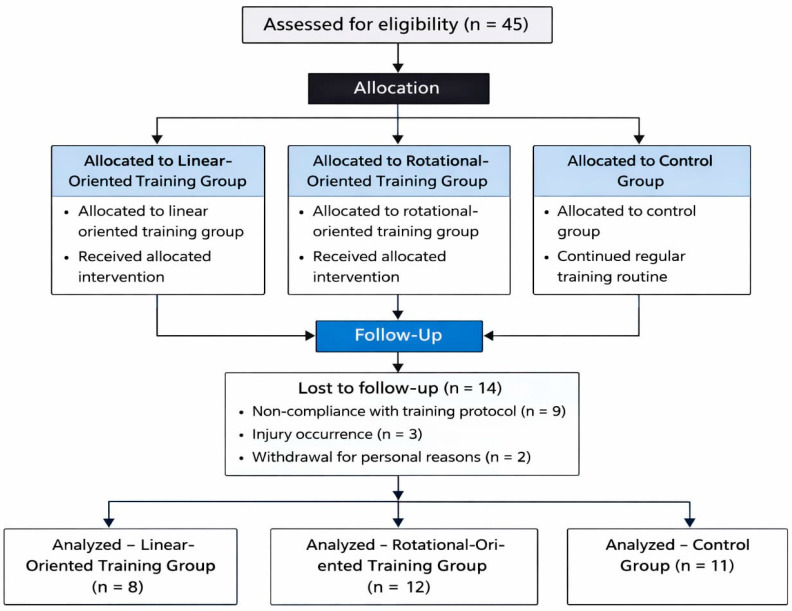
CONSORT (Consolidated Standards of Reporting Trials) flowchart.

An a priori power analysis (G*Power 3.1.9.7; Kiel University, Kiel, Germany) indicated that a total sample size of 36 participants was required based on the following parameters: repeated-measures ANOVA (within–between interaction), statistical power of β = 0.80, α level of 0.05, and an effect size of f = 0.25. Participants were eligible if they met the following criteria: at least one year of boxing experience, aged between 18 and 39 years, and a training frequency of at least three sessions per week for the past three months. Exclusion criteria included any injury or medical condition within the past 12 weeks that could interfere with training or testing. Consequently, 45 male boxing practitioners who responded to a written announcement posted at a local boxing club volunteered to participate in the study. However, only the 31 participants who completed the intervention were included in the statistical analyses and are therefore described in the baseline characteristics (age: 27.0 ± 6.1 years; years of practice: 3.3 ± 2.3 years). Fourteen participants did not complete the study due to non-compliance with the prescribed training protocol (*n* = 9), injury occurrence (*n* = 3), or withdrawal for personal reasons (*n* = 2). Dropout distribution across groups was as follows: seven participants from the LOTG, three from the ROTG, and four from the CG. The reported injuries were minor and related to regular boxing practice (e.g., hand inflammation due to impact during training) and were not associated with the experimental S&C interventions. The final sample consisted of 8 participants in the LOTG, 12 participants in the ROTG, and 11 participants in the CG (Figure 2). Due to this reduced sample size, an a priori power sensitivity analysis was completed based on *n* = 31, β = 0.80 and α = 0.05. This resulted in a minimum detectable effect size of f = 0.26. Following data analysis, a post hoc power analysis was undertaken to check the robustness of the reported results using the smallest recorded effect size that elicited a significant result (ω^2^ = 0.09). This found that the minimum post hoc power of the statistically significant results was β = 0.92, demonstrating sufficient power being retained despite a reduced sample.

Randomization was performed using a computer-generated sequence (www.randomizer.org; accessed on 25 October 2025) stratified by BM to ensure balanced allocation across groups. The allocation sequence was generated prior to baseline testing by a researcher not involved in outcome assessment. Due to the nature of the training interventions, participants and coaches could not be blinded to group assignment; however, data processing and statistical analyses were performed by investigators blinded to group identity.

### 2.4. Anthropometric and Body Composition

BH was measured to the nearest 0.1 cm using a stadiometer (Seca Model 217; Birmingham, UK), with participants standing barefoot. BM was assessed using an electronic scale (Seca 713; Hamburg, Germany) with a capacity of 150 kg and an accuracy of 100 g. Subsequently, body fat percentage (BF%) was assessed using a bioelectrical impedance analysis (BIA) device (Omron HBF-306C; Omron Healthcare, Kyoto, Japan). The Omron HBF-306C body composition monitor has demonstrated high reliability and a strong correlation with dual-energy X-ray absorptiometry (DXA), making it suitable for monitoring longitudinal changes in body composition [26]. The procedure strictly followed the manufacturer’s guidelines to ensure measurement reliability. Each participant was instructed to wear light sports clothing (shorts and a T-shirt) and to remove any metallic accessories prior to the evaluation. Participants maintained an upright posture and ensured full contact with the electrodes during the measurement. BMI was calculated as BMI = BM/BH^2^ (kg·m^−2^).

### 2.5. Measurement of Punch Impact Power

Punch impact power was measured using the PK (Strike Research Ltd., London, UK). Before the assessment, all participants received instructions and performed a familiarization session with the PK. This familiarization session was conducted prior to baseline testing to minimize potential learning effects and ensure consistent execution across subsequent assessment sessions. The procedures included the following: A general warm-up; Use of standardized gloves (10 oz) and bandages (2.5 m); Technical instructions on strike execution; A specific warm-up with shadow boxing; Individual adjustments of the PK to ensure the target was at chin height for precise measurement of both Straight punches and Hooks; Progressive effort tests to accustom participants to the instrument [27].

The general warm-up consisted of the following activities: 90 s of jumping rope, 10 forward and backward shoulder and elbow circles (each side), 10 front and side leg swings (each leg), 10 lunges per leg, 45 s of boxing footwork (front, back, left, right) on command. Participants received 3 min of supervised technical practice under a coach’s guidance. This included imitating the coach’s movements and receiving corrections to ensure proper technique. Coaches also identified and classified the type of punches delivered by each practitioner. Participants completed 3 rounds of shadow boxing, each lasting approximately 3 min, with 5 min of rest between rounds. Each round involved ~180 strikes (3 strikes every 3 s), executed in response to auditory signals from the coach.

The PK was individually adjusted for each participant and strike type. For Straight punches, participants assumed a standard boxing guard position, standing at a self-selected arm’s length from the PK, with the target at chin height. For Hook punches, participants stood next to the PK, with the fist in contact with the target at approximately 90° of elbow flexion.

For the maximal-effort condition, participants performed 6 Straight punches (3 Jabs [lead-hand Straight punches] and 3 Crosses [rear-hand Straight punches]) and 6 Hook punches with each hand, totaling 24 strikes. To prepare, they performed 4 strikes at 50% perceived maximum effort, followed by the maximum effort strikes. The order of strikes alternated between hands and punch types, with a minimum rest period of 3 s between punches to allow participants to regain proper positioning.

All assessment sessions (baseline, midpoint, and post-intervention) were conducted under identical testing procedures, standardized punch order, and equivalent recovery periods between trials to ensure consistency across measurements.

### 2.6. Power and Strength Muscle Tests

Participants performed the CMJ on an electronic contact platform (Chronojump Contact Platform A2, Bosco Systems, Barcelona, Spain) to measure the maximum vertical jump height. Athletes were instructed to place their hands on their hips and stand with their feet shoulder-width apart. They performed a countermovement by bending their knees until reaching a 90-degree angle before initiating the concentric phase of the CMJ. Three trials were completed, with 10–15 s of rest between them, and the best performance trial was used for the subsequent statistical analysis [28].

For the 1 RM BP test, normal standard Olympic bar and plates were used for the lifts. The bench press procedure was standard “touch-and-go” protocol [29]. Participants warmed up for the bench press test with 5 min of light cycling on a leg ergometer at a self-selected intensity. The specific warm-up began with 5 repetitions at approximately 50% of an estimated 1 RM BP, based on prior experience or evaluator guidance. After a 1-min rest, participants performed 3 repetitions with 60–70% of the estimated load. Following a 2 min rest, 2 repetitions were performed at 80–85% of the estimated load. Another 2 min rest preceded the first 1 RM BP attempt. The first attempt was performed with a load near 90–95% of the estimated 1 RM BP. If successful, the load was increased by 2–5% (minimum increase of weight was 3 kg); if unsuccessful, it was reduced by 2–5%. Participants were allowed 3–6 attempts to determine their 1 RM BP, depending on fatigue and success rates. Rest intervals of 3–5 min were provided between attempts to ensure recovery and minimize fatigue. A successful lift required the barbell to touch the chest, pause slightly, and be raised to full arm extension using correct technique [30,31].

Handgrip strength was assessed using a dynamometer (Takei Physical Fitness Test, TKK 5001, GRIP–A, Tokyo, Japan) to measure the strength of hand and forearm muscles. Participants were seated upright with hips and knees flexed at 90°, feet flat on the floor, and the tested arm positioned at the side without touching the torso. The elbow was flexed at 90°, forearm in a neutral position, and wrist positioned between 0° and 30° of extension, with 0° to 15° of ulnar deviation. Participants were instructed to squeeze the dynamometer with maximum force for 3–5 s, avoiding movement of the arm or trunk. The test was performed three times for each hand, with 60 s intervals between attempts to prevent fatigue. The highest recorded value from each hand was used for analysis [32,33].

This study was approved by the Local Ethics Committee of Lusófona University (approval number AB3025) on 30 April 2025, and all procedures were conducted in accordance with the Declaration of Helsinki for human experimentation [34]. Participants received detailed information about the study design, associated risks, and their right to withdraw at any stage of the process. Written informed consent was obtained from all participants before the commencement of the study.

### 2.7. Statistical Analyses

Data were analysed using IBM SPSS software (version 25.0; IBM Corp, Armonk, NY, USA). Normality of the data was assessed using the Shapiro–Wilk test. While most baseline and time-point variables showed approximately normal distributions, some Δ variables deviated from normality. However, although some variables showed deviations from normality, parametric analyses were retained, as analysis of variance (ANOVA) is considered robust to moderate violations of the normality assumption, particularly when sample sizes are similar across groups and homogeneity of variances is met [35]. Homogeneity of variances was assessed using Levene’s test. To further verify the robustness of the parametric analyses, additional non-parametric sensitivity analyses using Kruskal–Wallis tests were performed across all change-score (Δ) variables. Therefore, data are presented as mean ± standard deviation (SD). A per-protocol analytical approach was adopted to ensure that the reported outcomes reflected the effects of the intervention among participants who completed the training as prescribed. To verify the equivalence of groups at baseline, a one-way ANOVA was first conducted on all main outcome variables, confirming no significant differences between groups. Subsequently, to analyse the effects of the training programs on punch impact performance variables, a mixed-design repeated-measures ANOVA was employed. The model included Group (LOTG, ROTG, and CG) as the between-subjects factor, and Time (baseline, midpoint, and post-intervention) as the within-subjects factor. This approach enabled the examination of main effects for Group and Time, as well as the Group × Time interaction, which was considered the primary analysis for testing the study hypotheses. When the assumption of sphericity was violated, Greenhouse–Geisser corrections were applied. Significant main effects or interaction effects were further explored using the Tukey HSD post hoc test.

To complement the primary interaction analyses, absolute change scores (Δ = post − pre) were calculated for each punch variable to further compare the magnitude of training-induced changes between groups. Between-group differences in Δ values were explored using one-way analysis of variance (one-way ANOVA). Pairwise comparisons were performed using the Tukey HSD post hoc test when significant main effects were observed.

Effect sizes were reported as omega squared (ω^2^), which provides a less biased estimate of the proportion of variance explained in ANOVA models [36,37]. Effect sizes (ω^2^) were interpreted using commonly applied benchmarks: <0.01 very small, 0.01–0.06 small, 0.06–0.14 medium, and ≥0.14 large [38].

No additional global correction for multiple testing was applied, as analyses were based on predefined outcome variables and repeated-measures ANOVA models with Tukey HSD post hoc procedures inherently controlled for multiple comparisons within each model.

Statistical significance was accepted when *p* < 0.05.

## 3. Results

Baseline analyses using one-way ANOVA revealed no significant between-group differences for anthropometric and performance variables (BM, BF%, BH, CMJ, HS, and 1 RM BP; *p* > 0.05), indicating adequate initial comparability between groups (Table 4). Detailed pre- and post-intervention values for anthropometric and performance variables across groups are provided in Supplementary Table S1.

A significant main effect of time was observed for all punch types (*p* < 0.001), indicating overall improvements in punch impact power across the intervention period. A significant main effect of group was detected only for the Jab (*p* = 0.035), whereas no significant group effects were found for the Cross or the Hook punches. Significant time × group interactions were identified for the Cross (*p* = 0.027), Lead Hook (*p* = 0.009), and Rear Hook (*p* < 0.001), indicating that the magnitude of improvement over time differed between training groups for these punches. No significant time × group interaction was observed for the Jab (*p* = 0.074) (Table 5).

In the between-group analyses of punch impact power improvements (post–pre), one-way ANOVA revealed a significant group effect for the Jab (*p* = 0.049), Cross (*p* = 0.039), Lead Hook (*p* = 0.005), and Rear Hook (*p* < 0.001) (Table 6). Although some Δ variables deviated from normality according to the Shapiro–Wilk test, one-way ANOVA was retained due to its robustness to moderate violations of normality in balanced designs. Homogeneity of variances was satisfied, as confirmed using Levene’s test. To further verify the robustness of these findings, additional non-parametric sensitivity analyses using Kruskal–Wallis tests were conducted. These analyses confirmed significant between-group differences for Lead Hook performance (H = 10.523, *p* = 0.005), while similar directional trends were observed for Jab (H = 5.227, *p* = 0.073), Cross (H = 5.563, *p* = 0.062), and Rear Hook performance (*p* = 0.062), with the overall pattern of results remaining consistent with the parametric analyses.

**Figure 3:**
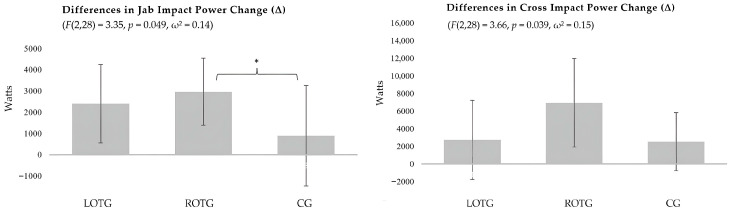

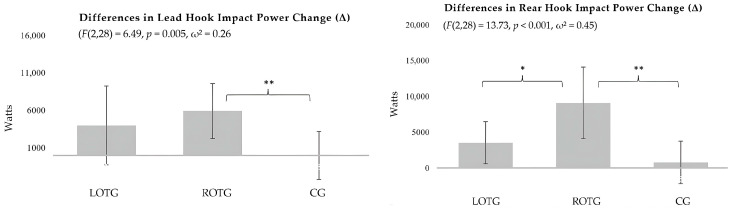
Between-group differences in punch impact power change (Δ) for the Jab, Cross, Lead Hook, and Rear Hook. Bars represent mean ± SD of post–pre changes. One-way ANOVA was used to compare groups, with corresponding *F*, *p*, and ω^2^ values reported in each panel. Significant pairwise differences identified using Tukey HSD post hoc tests (* *p* < 0.05, ** *p* < 0.01). LOTG = Linear-Oriented Training Group; ROTG = Rotational-Oriented Training Group; CG = Control Group.

Post hoc Tukey tests revealed that ROTG showed significantly greater improvements than a CG for the Jab (*p* = 0.044) and Lead Hook (*p* = 0.004), and greater improvements than both LOTG (*p* = 0.011) and CG (*p* < 0.001) for the Rear Hook (Figure 3).

A significant main effect of time was observed for all strength and power variables, including the CMJ (*p* < 0.001), HS (*p* < 0.001), and 1 RM BP (*p* < 0.001), indicating overall improvements across the intervention period. A significant main effect of group was found only for the 1 RM BP (*p* = 0.002), whereas no significant group effects were observed for the CMJ (*p* = 0.334) or HS (*p* = 0.081). Significant time × group interactions were identified for all variables, namely CMJ (*p* = 0.021), HS (*p* = 0.020), and 1 RM BP (*p* < 0.001), indicating that the magnitude of changes over time differed between training groups for these measures (Table 7).

In the between-group analyses of strength and power improvements (post–pre), one-way ANOVA revealed significant group effects for the CMJ (*p* = 0.017), HS (*p* = 0.016), and 1 RM BP (*p* < 0.001) (Table 8).

## 4. Discussion

The purpose of this study was to examine the effects of targeted S&C interventions on punch impact power in three groups of boxers, comparing programs emphasizing linear force production (LOTG), rotational and lateral force production (ROTG), and a regular boxing training approach (CG). The main findings partially support the study hypotheses, demonstrating that punch-specific training programs elicited greater improvements in punch impact power than conventional training alone, with adaptations that were largely specific to punch type and training focus.

Punch impact power increased across the intervention period regardless of group. This finding suggests that regular boxing practice, combined with structured training over eight weeks, contributes to overall improvements in punching performance. Such improvements are consistent with previous research showing that repeated exposure to high-intensity boxing-specific actions enhances neuromuscular coordination and force transmission along the kinetic chain [13,14,15]. From a mechanistic perspective, these improvements may reflect enhanced intermuscular coordination and more effective force transfer during punching actions.

However, the presence of significant group × time interactions for the Cross, Lead Hook, and Rear Hook indicates that the magnitude of improvement over time differed between training groups. These interaction effects represent the primary statistical basis for interpreting between-group differences in training adaptations across the intervention period. From an applied perspective, they further indicate that not all training approaches produce equivalent adaptations and that activity-specific S&C can influence performance outcomes beyond what boxing training alone can achieve. From a physiological perspective, S&C training likely provides neuromuscular stimuli that are not fully elicited through boxing practice alone. These include increases in maximal force production, rate of force development, and motor unit recruitment and synchronization [16]. Additionally, structured strength and power exercises may enhance force application in lateral and rotational movement patterns, as well as improve trunk and lower-limb force transmission, as suggested by recent biomechanical analyses examining effective mass and segmental acceleration contributions to punching performance [39]. These adaptations may allow athletes to apply force more rapidly during punching actions. This may help explain the superior improvements observed in groups exposed to targeted S&C interventions.

Between-group analyses of absolute changes (Δ values) were conducted as complementary analyses to further clarify the magnitude of the interaction effects identified in the mixed-design ANOVA. Significant group differences were observed for all punch types, with the largest effect sizes found for the Hook punches, particularly the Rear Hook (ω^2^ = 0.47) (Table 5). These findings may be explained by the greater biomechanical and neuromuscular demands of Hook punches, which rely heavily on trunk rotation and coordinated force transfer through the torso and lower limbs, as previously described in boxing biomechanics literature [1,11].

Our first hypothesis was not supported. The LOTG, which emphasized linear force trajectories and force transfer from the lower limbs through the trunk to the upper extremities, did not produce greater improvements in punch impact power compared with the other groups. Although some gains were observed, these were not statistically significant relative to the CG and were smaller than those achieved by the ROTG, including for the Straight punch. While Straight punches are traditionally characterized by predominantly linear force transmission [10], the adaptations induced by the LOTG may have been insufficient to generate a clear performance advantage. In contrast, the superior improvements observed in the ROTG suggest that rotational and lateral force production may play a more influential role, even in punches commonly classified as linear. Rotational contributions from the trunk and pelvis may positively influence the acceleration process by increasing distal segment acceleration, thereby enhancing impact power [39]. This interpretation is consistent with the importance of rotational components in Straight punches reported in previous research [9,10].

In contrast, our findings supported the second hypothesis. The ROTG consistently demonstrated the greatest improvements in Hook punch impact power, supporting the hypothesis that training programs emphasizing rotational and lateral force production are particularly effective for enhancing Hook performance. The significant difference between ROTG and LOTG illustrated in Figure 3 indicates that the ROTG achieved greater improvements in Rear Hook impact power than the LOTG. Overall, these results indicate that training programs aligned with the biomechanical requirements of Hook punches—rotational and lateral force production—rather than purely linear force production, can induce substantial increases in Hook impact power [11,15]. Additionally, the Hook punches exhibited the highest absolute impact power values, aligned with recent research, which likely amplified between-group differences and contributed to the observed statistical significance [21]. From an applied perspective, these improvements in Hook punch impact power may be particularly meaningful in competitive boxing contexts, as increases in rotationally generated striking force may enhance the likelihood of earlier bout termination through knockout, which represents one key performance objective for athletes [3]. Unlike Straight punches, Hooks involve less elbow extension and greater trunk rotation, which may increase proximal stiffness and reduce energy dissipation through the upper limb and enhance higher force transmission to the target [39,40,41].

The third hypothesis was confirmed: the CG consistently exhibited smaller improvements across all punch types, suggesting that traditional boxing training alone—while effective for maintaining and modestly improving performance—may not optimally develop the underlying physical determinants of punch impact power. This observation supports previous critiques of conventional boxing training models that prioritize technical repetition and sparring without sufficient emphasis on biomechanically targeted resistance training [15]. It is therefore plausible that S&C exercises emphasizing punch-specific mechanics—particularly those involving rotational movements and substantial trunk engagement—implemented across the intervention groups promoted greater strength and power adaptations, resulting in enhanced performance across several punch-specific outcomes, particularly Hook punches. These findings highlight the importance of targeted S&C interventions in eliciting superior mechanical and neuromuscular adaptations compared with regular boxing training alone. Such adaptations likely arise from the exposure to upper-body ballistic exercises, plyometric actions, resistance training, and rotational-focused movements, which provide stimuli for strength, power, and neuromuscular coordination that are typically absent or insufficiently developed during standard boxing training sessions [15,16,41].

In addition to punch-specific outcomes, our findings supported the fourth hypothesis, as participants in the LOTG and ROTG demonstrated greater improvements in general strength and power, assessed using the CMJ, HS, and 1 RM BP. These findings are further supported by the mixed-design ANOVA results, which revealed significant main effects of time for all general strength and power variables, indicating overall improvements across the intervention period. Importantly, significant time × group interactions were observed for CMJ, HS, and 1 RM BP, demonstrating that the magnitude and progression of adaptations differed between training groups over time for all measures. However, a significant main effect of group was identified only for the 1 RM BP, indicating consistent between-group differences in upper-body maximal strength irrespective of time, whereas group differences in CMJ and HS were time-dependent. Collectively, these results suggest that while all training approaches promoted general performance improvements, only bench press strength adaptations showed stable between-group differences, whereas changes in lower-body power and grip strength were more sensitive to the temporal dynamics of the intervention.

The between-group analysis of change scores further clarifies these adaptations, as significant differences were observed between groups for CMJ, HS, and 1 RM BP. Specifically, the LOTG demonstrated greater improvements in CMJ compared with the CG, whereas the ROTG showed intermediate values that did not differ significantly from either group. No significant pairwise differences were identified between groups for HS in post hoc comparisons. While the most pronounced between-group differences were evident for the 1 RM BP, with the LOTG exhibiting the greatest absolute gains, complementary between-group analyses of change scores (post–pre) helped to further interpret the magnitude of these adaptations across groups. These superior improvements in 1 RM BP observed in the LOTG may be attributed, at least in part, to the inclusion of the bench press exercise within their training program, which provided a highly specific mechanical and neuromuscular stimulus aligned with the assessment task. According to the principle of training specificity, early and task-dependent strength adaptations are largely driven by neural mechanisms that are specific to the trained movement pattern, joint configuration, and loading characteristics [42]. Consequently, repeated exposure to the bench press likely enhanced neural drive, motor unit recruitment, and intermuscular coordination in a manner that preferentially improved performance in that exercise. However, when interpreted alongside the punch-specific outcomes, these findings further highlight a dissociation between the magnitude of general strength gains and their transfer to punching performance. Despite achieving the largest improvements in maximal upper-body strength, the LOTG did not demonstrate the greatest increases in punch impact power, whereas the ROTG exhibited superior punch-specific adaptations. This reinforces the notion that while maximal strength development is an important physical quality, task specificity, force–time characteristics, and movement coordination appear to be more decisive factors for optimizing punching performance.

Taken together, the results presented in Table 7 and Table 8 demonstrate that the training interventions induced distinct and meaningful neuromuscular adaptations between groups, particularly favoring the intervention groups. The mixed-design ANOVA interaction effects provided the primary statistical evidence for these differential training responses, whereas change-score analyses offered complementary information regarding the magnitude of these adaptations. Specifically, while the mixed-design ANOVA revealed significant time × group interactions for CMJ and HS, the between-group analysis of change scores (post–pre) further clarified these findings by demonstrating significant differences in CMJ improvements, with the LOTG exhibiting greater gains than the CG, whereas no significant pairwise differences were observed between groups for HS. These results indicate that structured S&C training promoted superior adaptations in lower-body power and grip strength beyond those achieved through regular boxing practice alone. Similarly, both intervention groups demonstrated markedly greater improvements in upper-body maximal strength, as reflected by larger gains in 1 RM BP. Moreover, these neuromuscular performance indicators highlight their relevance to punching performance, as increases in these variables within the intervention groups occurred concurrently with improvements in punch impact power. Together, these adaptations reinforce the notion that punch effectiveness is not solely a technical skill but also a manifestation of whole-body neuromuscular performance. However, although the superior punch-specific adaptations observed in the ROTG are consistent with the principle of biomechanical specificity, alternative explanations such as differences in exercise novelty or participant engagement cannot be completely excluded and should be considered when interpreting the magnitude of these adaptations, particularly given that direct assessments of neuromuscular activation patterns (e.g., surface electromyography) were not performed, which have been shown to provide important insight into striking movement efficiency in recent sport-specific investigations [43].

## 5. Limitations

Several limitations should be acknowledged. First, although the a priori power analysis indicated that 36 participants were required, the final analyzed sample (*n* = 31) was slightly smaller than originally planned due to participant dropout, which may have reduced statistical power relative to the original design assumptions, and slightly increased the minimum detectable effect size. The size of the effects reported, however, led to power being increased in post hoc analyses. The unequal number of participants per group, however, may have limited the detection of smaller yet practically meaningful differences between groups. Therefore, results should be interpreted with caution, and future studies with larger samples are warranted to strengthen generalizability. Although a per-protocol approach was adopted to ensure that analyses reflected the effects of completed interventions, the exclusion of participants who did not complete the training protocol may have introduced a potential risk of attrition bias. Second, the sample included male boxing practitioners with heterogeneous training backgrounds, which may have increased inter-individual variability and limits extrapolation to elite, novice, or female athletes. Third, all participants were recruited from the same training environment, which enhanced internal consistency but may restrict external validity. Future studies should include athletes from diverse training contexts. Fourth, punch performance was assessed exclusively through impact power outcomes. Although the measurement system demonstrated high reliability, the absence of detailed biomechanical analyses limits mechanistic interpretation. Specifically, variables related to force transmission along the kinetic chain, trunk rotational mechanics (e.g., trunk rotation velocity derived from 3D kinematic analyses), proximal stiffness, and neuromuscular recruitment (e.g., electromyographic activity) were not directly measured, and therefore the mechanistic explanations presented in the Discussion should be interpreted as literature-supported interpretations rather than direct evidence from the present study. Additionally, although external training load was controlled through intensity prescription and progression criteria, accumulated training volume and internal load measures (e.g., session-RPE) were not systematically monitored, which may limit detailed comparisons of workload between groups and should be addressed in future research. Finally, the relatively short intervention period may not reflect longer-term adaptations or plateau effects. Extended longitudinal studies are warranted.

## 6. Conclusions

This study demonstrates that targeted S&C interventions elicit punch-specific adaptations in boxing practitioners. Contrary to our initial hypothesis, programs emphasizing linear force production did not produce superior improvements in Straight punch impact power compared with rotational and lateral-focused training. Notably, rotational-oriented training produced greater improvements, particularly in Hook punch impact power and selected punch-specific outcomes. These findings support the integration of biomechanically specific resistance training into boxing preparation and highlight the importance of aligning strength development with the mechanical demands of different punch actions.

## 7. Practical Implications

The findings provide actionable guidance for boxing coaches and S&C professionals. Punch-specific resistance training that emphasizes multi-planar force production—particularly rotational and lateral movements—appears effective for improving impact power, especially in Hook punches and selected punch-specific performance outcomes. Coaches should therefore prioritize exercises reflecting the multi-directional nature of punching mechanics, such as rotational medicine ball throws, landmine variations, and cable-based drills. Importantly, these interventions should complement, not replace, traditional boxing training. Integrating biomechanically aligned strength and power exercises with technical practice may enhance punch performance while maintaining skill development.

## Figures and Tables

**Table 1:** Training Program Week.

Day of the Week	Training Week
Monday	AM: S&C (LOTG, ROTG); PM: Boxing (LOTG, ROTG, CG)
Tuesday	PM: Boxing (LOTG, ROTG, CG)
Wednesday	PM: Boxing (LOTG, ROTG, CG)
Thursday	PM: Boxing (LOTG, ROTG, CG)
Friday	AM: S&C (LOTG, ROTG); PM: Boxing (LOTG, ROTG, CG)
Saturday	Off
Sunday	Off

Notes: LOTG = Linear-Oriented Training Group; ROTG = Rotational-Oriented Training Group; CG = Control Group; S&C = Strength and Conditioning.

**Table 2:** Monday Training Program.

Strength & Power Exercises	**Exercise**	**Sets × Reps × Load**	**Rest**
	Mobility Warm Up (LOTG, ROTG).	5 min	
	- Hexagonal Bar Deadlift (LOTG) - Landmine Lateral Lunge (ROTG)	4 × 5 × 85% of 1 RM	3 min
	- Jump Lunges (LOTG) - Side Squat (ROTG)	3 × 8	3 min
	- One Max Forward Jump (LOTG). - One Max Side Jump (ROTG)	3 × 8	3 min
	- Barbell Bench Press (LOTG) - Dumbbell Chest Flys (ROTG)	4 × 5 × 85% of 1 RM	3 min
	- Straight Punch Medicine Ball Throws (LOTG). - Hook Punch Medicine Ball Throws (ROTG).	2 × 8 × 3 kg	3 min
	- Cable Row (LOTG) - Unilateral Cable Row (ROTG).	4 × 5 × 85% of 1 RM	3 min

Notes: LOTG = Linear-Oriented Training Group; ROTG = Rotational-Oriented Training Group; 1 RM = 1 repetition maximum.

**Table 3:** Friday Training Program.

Strength & Power Exercises	**Exercise**	**Sets × Reps × Load**	**Rest**
	Mobility Warm Up (LOTG, ROTG).	5 min	
	- Hexagonal Bar Deadlift (LOTG) - Landmine Lateral Lunge (ROTG)	4 × 5 × 85% of 1 RM	3 min
	- Straight Punch Medicine Ball Throws with Front Step (LOTG). - Hook Punch Medicine Ball Throws with Lateral Step (ROTG).	3 × 8 × 3 kg	3 min
	- Barbell Bench Press (LOTG) - Dumbbell Chest Flys (ROTG)	4 × 5 × 85% of 1 RM	3 min
	- Landmine Straight Punch with 10% BM (LOTG) - Unilateral Hooks with 20 kg Elastic Bend (ROTG).	3 × 8	3 min
	- Isometric Straight Punch (LOTG). - Isometric Hook Punch (ROTG).	3 × 30 seg each hand	3 min
	- Two Repeated Forward Jumps (LOTG). - Two Repeated Side Jumps (ROTG).	3 × 8	3 min

Notes: LOTG = Linear-Oriented Training Group; ROTG = Rotational-Oriented Training Group; 1 RM = 1 repetition maximum.

**Table 4:** Baseline characteristics of participants included in the final analysis (mean ± SD) with between-group comparisons identified via one-way ANOVA.

Variable	LOTG (*n* = 8)	ROTG (*n* = 12)	CG (*n* = 11)	*p*
Body mass (kg)	77.24 ± 1.99	75.22 ± 2.92	76.02 ± 3.27	0.371
Body fat (%)	21.90 ± 4.19	17.52 ± 7.05	22.77 ± 5.50	0.09
Body height (cm)	177.15 ± 3.87	175.57 ± 6.50	174.36 ± 8.69	0.68
CMJ (cm)	33.76 ± 4.59	34.07 ± 7.18	32.31 ± 4.91	0.75
HS (kg)	45.54 ± 4.02	43.09 ± 2.81	43.49 ± 4.02	0.21
1 RM BP (kg)	85.63 ± 12.66	78.75 ± 10.90	71.82 ± 5.61	0.14

Notes: CMJ = countermovement jump; HS = handgrip strength; 1 RM BP = one repetition maximum bench press. No significant between-group differences were observed at baseline (*p* > 0.05).

**Table 5:** Main effects of time and the group × time interactions obtained from the mixed-design repeated-measures ANOVA for all punch impact variables.

Variable	Effect	*F*_(df1, df2)_	*p*	ω^2^
Jab	Time	*F*_(2, 56)_ = 20.93	<0.001 ***	0.37
	Group	*F*_(2, 28)_ = 3.79	0.035 *	0.14
	Time × Group	*F*_(4, 56)_ = 2.26	0.074	0.04
Cross	Time	*F*_(1.35, 37.87)_ = 21.33	<0.001 ***	0.36
	Group	*F*_(2, 28)_ = 0.53	0.596	0.00
	Time × Group	*F*_(2.71, 37.87)_ = 3.54	0.027 *	0.09
Lead Hook	Time	*F*_(1.25, 35.01)_ = 12.77	<0.001 ***	0.28
	Group	*F*_(2, 28)_ = 0.31	0.738	0.00
	Time × Group	*F*_(2.51, 35.01)_ = 4.90	0.009 **	0.20
Rear Hook	Time	*F*_(1.34, 37.61)_ = 24.84	<0.001 ***	0.47
	Group	*F*_(2, 28)_ = 0.25	0.781	0.08
	Time × Group	*F*_(2.68, 37.61)_ = 8.97	<0.001 ***	0.34

Notes: *F* = *F* statistic from the mixed-design repeated-measures ANOVA; df1 and df2 = numerator and denominator degrees of freedom; *p* = significance level; ω^2^ = omega squared effect size; * *p* < 0.05; ** *p* < 0.01; *** *p* < 0.001. Cross = Rear-hand Straight Punch; Jab = Lead-hand Straight Punch.

**Table 6:** Between-group differences in punch performance improvements (Δ values) identified via one-way ANOVA.

Variable (Δ)	LOTG (*n* = 8)	ROTG (*n* = 12)	CG (*n* = 11)	*F*_(df1, df2)_	*p*	ω^2^
Jab (W)	2399 ± 1852 ^ab^	2961 ± 1582 ^a^	888 ± 2372 ^b^	*F*_(2, 28)_ = 3.35	0.049 *	0.13
Cross (W)	2739 ± 4503 ^a^	6952 ± 5031 ^a^	2529 ± 3301 ^a^	*F*_(2, 28)_ = 3.66	0.039 *	0.15
Lead Hook (W)	4002 ± 5263 ^ab^	5911 ± 3671 ^a^	−16 ± 3217 ^b^	*F*_(2, 28)_ = 6.49	0.005 **	0.26
Rear Hook (W)	3532 ± 2930 ^b^	9097 ± 4971 ^a^	774 ± 2978 ^b^	*F*_(2, 28)_ = 13.73	<0.001 ***	0.45

Notes: Δ values represent post–pre changes; *F* = *F* statistic from one-way ANOVA; df1 and df2 = degrees of freedom; *p* = significance level; ω^2^ = omega squared effect size; * *p* < 0.05; ** *p* < 0.01; *** *p* < 0.001. Different superscript letters indicate significant differences between groups based on Tukey HSD post hoc comparisons. LOTG = Linear-Oriented Training Group; ROTG = Rotational-Oriented Training Group; CG = Control Group; Cross = Rear-hand Straight Punch; Jab = Lead-hand Straight Punch.

**Table 7:** Main effects of time and the group × time interactions obtained from the mixed-design repeated-measures ANOVA for all strength and power variables.

Variable	Effect	*F*_(df1, df2)_	*p*	ω^2^
CMJ	Time	*F*_(1.49, 41.69)_ = 13.90	<0.001 ***	0.30
	Group	*F*_(2, 28)_ = 1.14	0.334	0.09
	Time × Group	*F*_(2.98, 41.69)_ = 3.63	0.021 *	0.15
HS	Time	*F*_(2, 56)_ = 19.32	<0.001 ***	0.38
	Group	*F*_(2, 28)_ = 2.75	0.081	0.10
	Time × Group	*F*_(4, 56)_ = 3.19	0.020 *	0.09
1 RM BP	Time	*F*_(1.13, 31.63)_ = 41.17	<0.001 ***	0.46
	Group	*F*_(2, 28)_ = 7.91	0.002 **	0.31
	Time × Group	*F*_(2.26, 31.63)_ = 8.30	<0.001 ***	0.17

Notes: *F* = *F* statistic from the mixed-design repeated-measures ANOVA; df1 and df2 = numerator and denominator degrees of freedom; *p* = significance level; ω^2^ = omega squared effect size; * *p* < 0.05; ** *p* < 0.01; *** *p* < 0.001. CMJ = countermovement jump; HS = handgrip strength; 1 RM BP: 1 repetition maximum bench press.

**Table 8:** Between-group differences in strength and power improvements (Δ values) identified via one-way ANOVA.

Variable (Δ)	LOTG (*n* = 8)	ROTG (*n* = 12)	CG (*n* = 11)	*F*_(df1, df2)_	*p*	ω^2^
CMJ	3.08 ± 2.11 ^b^	2.82 ± 3.32 ^ab^	0.01 ± 1.68 ^a^	*F*_(2, 28)_ = 4.69	0.017 *	0.19
HS	4.45 ± 2.00 ^a^	3.88 ± 3.88 ^a^	0.64 ± 2.44 ^a^	*F*_(2, 28)_ = 4.83	0.016 *	0.20
1 RM BP	20.63 ± 7.89 ^b^	10.83 ± 12.40 ^ab^	2.27 ± 5.64 ^a^	*F*_(2, 28)_ = 9.20	<0.001 ***	0.37

Notes: Δ values represent post–pre changes. *F* = *F* statistic from one-way ANOVA; df1 and df2 = degrees of freedom; *p* = significance level; ω^2^ = omega squared effect size; * *p* < 0.05; *** *p* < 0.001. Different superscript letters indicate significant differences between groups based on Tukey HSD post hoc comparisons. LOTG = Linear-Oriented Training Group; ROTG = Rotational-Oriented Training Group; CG = Control Group; CMJ = countermovement jump; HS = handgrip strength; 1 RM BP = 1 repetition maximum bench press.

## Data Availability

Data generated or analyzed during this study are available from the corresponding author upon reasonable request.

## Supplementary Materials

The following supporting information can be downloaded at: https://www.mdpi.com/article/10.3390/sports14050194/s1, Table S1: Pre- and post-intervention values of anthropometric and performance variables across groups (mean ± SD).

## References

[1] Hristovski R., Davids K., Araújo D., Button C. (2006). How boxers decide to punch a target: Emergent behaviour in nonlinear dynamical movement systems. J. Sports Sci. Med..

[2] Pinto M., Monteiro L. (2025). Traducción al portugués del instrumento “plantilla de análisis del desempeño del boxeo”. Retos.

[3] Beattie K., Ruddock A.D. (2022). The role of strength on punch impact force in boxing. J. Strength Cond. Res..

[4] Chaabène H., Tabben M., Mkaouer B., Franchini E., Negra Y., Hammami M., Amara S., Chaabène R.B., Hachana Y. (2015). Amateur boxing: Physical and physiological attributes. Sports Med..

[5] Kim K.J., Lee S.B., Park S. (2018). Effects of boxing-specific training on physical fitness and punch power in Korean national boxers. Exerc. Sci..

[6] Del Vecchio L., Whitting J., Hollier J., Keene A., Climstein M. (2022). Reliability and practical use of a commercial device for measuring punch and kick impact kinetics. Sports.

[7] Lenetsky S., Harris N.K., Brughelli M. (2013). Assessment and contributors of punching forces in combat sports athletes: Implications for strength and conditioning. Strength Cond. J..

[8] Pinto M., Crisóstomo J., Kirk C., Abián-Vicén J., Monteiro L. (2025). Influence of anthropometric characteristics and muscle performance on punch impact. Sports.

[9] Filimonov V.I., Koptsev K.N., Husyanov Z.M., Nazarov S.S. (1985). Boxing: Means of increasing strength of the punch. Natl. Strength Cond. Assoc. J..

[10] Piorkowski B.A., Lees A., Barton G.J. (2011). Single maximal versus combination punch kinematics. Sports Biomech..

[11] Bingul B.M., Bulgun C., Tore O., Bal E., Aydin M. (2018). The effects of biomechanical factors to teach different hook punch techniques in boxing and education strategies. J. Educ. Train. Stud..

[12] Kostikiadis I.N., Methenitis S., Tsoukos A., Veligekas P., Terzis G., Bogdanis G.C. (2018). The effect of short-term sport-specific strength and conditioning training on physical fitness of well-trained mixed martial arts athletes. J. Sports Sci. Med..

[13] Ojeda-Aravena A., Herrera-Valenzuela T., Valdés-Badilla P., Báez-San Martín E., Thapa R.K., Ramírez-Campillo R. (2023). A systematic review with meta-analysis on the effects of plyometric-jump training on the physical fitness of combat sport athletes. Sports.

[14] Matthews M., Comfort P. (2008). Applying complex training principles to boxing: A practical approach. Strength Cond. J..

[15] Ruddock A.D., Wilson D.C., Thompson S.W., Hembrough D., Winter E.M. (2016). Strength and conditioning for professional boxing: Recommendations for physical preparation. Strength Cond. J..

[16] Uthoff A., Lenetsky S., Reale R., Falkenberg F., Pratt G., Amasinger D., Bourgeois F., Cahill M., French D., Cronin J. (2023). A review of striking force in full-contact combat sport athletes. Strength Cond. J..

[17] Suchomel T.J., Nimphius S., Stone M.H. (2016). The importance of muscular strength in athletic performance. Sports Med..

[18] Dunn E.C., Humberstone C.E., Franchini E., Iredale K.F., Blazevich A.J. (2022). Relationships between punch impact force and muscular strength and power in amateur boxers. J. Strength Cond. Res..

[19] López-Laval I., Sitko S., Muñiz-Pardos B., Cirer-Sastre R., Calleja-González J. (2020). Relationship between bench press strength and punch performance in male professional boxers. J. Strength Cond. Res..

[20] Loturco I., Pereira L.A., Kobal R., Fernandes V., Reis V.P., Romano F., Alves M., Freitas T., McGuigan M. (2021). Transference effect of short-term optimum power load training on punching impact. J. Strength Cond. Res..

[21] Pinto M., Crisóstomo J., Silva G., Monteiro L. (2025). The influence of anthropometric characteristics on punch impact. Sports.

[22] Finlay M.J. (2022). World heavyweight championship boxing: The past 30+ years of the male division. PLoS ONE.

[23] Akın B., Koçoğlu-Tanyer D. (2021). SPIRIT 2013 bildirisi: Klinik deneyler için standart protokol maddelerinin tanımlanması. Hacettepe Univ. Hemşirelik Fak. Derg..

[24] Tunn R., Boutron I., Chan A.W., Collins G.S., Hróbjartsson A., Moher D., Schulz K.F., de Beyer J.A., Nejstgaard C.H., Østengaard L. (2024). Methods used to develop the SPIRIT 2024 and CONSORT 2024 statements. J. Clin. Epidemiol..

[25] Blair S.N., Haskell W.L., Ho P., Paffenbarger R.S., Vranizan K.M., Farquhar J.W., Wood P.D. (1985). Assessment of habitual physical activity by a seven-day recall. Am. J. Epidemiol..

[26] Cruz Rivera P.N., Goldstein R.L., Polak M., Lazzari A.A., Moy M.L., Wan E.S. (2022). Performance of bioelectrical impedance analysis compared to DXA in veterans with COPD. Sci. Rep..

[27] Omcirk D., Vetrovsky T., Padecky J., Malecek J., Tufano J.J. (2023). Validity of commercially available punch trackers. J. Strength Cond. Res..

[28] Ramírez-Campillo R., Andrade D.C., Izquierdo M. (2013). Effects of plyometric training volume and training surface on explosive strength. J. Strength Cond. Res..

[29] Mayhew J.L., Ware J.S., Cannon K., Corbett S., Chapman P.P., Bemben M.G., Ward T.E., Farris B., Juraszek J., Slovak J.P. (2002). Validation of the NFL-225 test for predicting 1-RM bench press performance in college football players. J. Sports Med. Phys. Fitness.

[30] Baechle T.R., Earle R.W. (2008). Essentials of Strength Training and Conditioning.

[31] Rontu J.P., Hannula M.I., Leskinen S., Linnamo V., Salmi J.A. (2010). One-repetition maximum bench press performance estimated with a new accelerometer method. J. Strength Cond. Res..

[32] MacDermid J., Solomon G., Valdes K. (2015). Clinical Assessment Recommendations.

[33] Myles L., Massy-Westropp N., Barnett F. (2024). The how and why of handgrip strength assessment. Br. J. Occup. Ther..

[34] World Medical Association (2013). World Medical Association declaration of Helsinki: Ethical principles for medical research involving human subjects. JAMA.

[35] Schmider E., Ziegler M., Danay E., Beyer L., Bühner M. (2010). Is it really robust? Reinvestigating the robustness of ANOVA against violations of the normality assumption. Methodology.

[36] Lakens D. (2013). Calculating and reporting effect sizes to facilitate cumulative science. Front. Psychol..

[37] Olejnik S., Algina J. (2003). Generalized eta and omega squared statistics. Psychol. Methods.

[38] Ben-Shachar M., Lüdecke D., Makowski D. (2020). effectsize: Estimation of effect size indices and standardized parameters. J. Open Source Softw..

[39] Mosler D., Kacprzak J., Wąsik J. (2024). The influence of effective mass on the striking force of lead jab and rear cross punches of boxers. Appl. Sci..

[40] Kacprzak J., Mosler D., Tsos A., Wąsik J. (2025). Biomechanics of punching—The impact of effective mass and force transfer on strike performance. Appl. Sci..

[41] Lee B., McGill S. (2017). The effect of core training on distal limb performance during ballistic strike manoeuvres. J. Sports Sci..

[42] Sale D.G. (1988). Neural adaptation to resistance training. Med. Sci. Sports Exerc..

[43] Csákvári L., Kopper B., Horváth T. (2025). A Novel, Sport-Specific EMG-Based Method to Evaluate Movement Efficiency in Karate Punching. Sports.

