# Supplementary material for: Effects of Specific Training Programs on Punch Performance
Source: Sports (Basel). 2026 May 8;14(5):194. doi: 10.3390/sports14050194 (PMC13211055; doi:10.3390/sports14050194)
Supplement: Supplementary file 1 [file sports-14-00194-s001.zip › sports-4266979-supplementary.pdf]

Supplementary Table S1. Pre- and post-intervention values of anthropometric and performance variables across groups (mean ± SD).

Variable	LOTG Pre (n = 8)	LOTG Post (n = 8)	ROTG Pre (n = 12)	ROTG Post (n = 12)	CG Pre (n = 11)	CG Post (n = 11)
Body mass (kg)	77.24 ± 1.99	77.74 ± 1.62	75.22 ± 2.92	75.75 ± 2.62	76.02 ± 3.27	76.11 ± 4.14
Body fat (%)	21.90 ± 4.19	19.73 ± 2.26	17.52 ± 7.05	18.13 ± 5.86	22.77 ± 5.50	22.50 ± 5.05
Body height (cm)	177.15 ± 3.87	177.15 ± 3.87	175.57 ± 6.50	175.57 ± 6.50	174.36 ± 8.69	174.36 ± 8.69
CMJ (cm)	33.76 ± 4.59	36.85 ± 4.96	34.07 ± 7.18	36.88 ± 6.52	32.31 ± 4.91	32.33 ± 5.27
HS (kg)	45.54 ± 4.02	49.99 ± 5.70	43.09 ± 2.81	46.95 ± 2.56	43.49 ± 4.02	44.13 ± 4.52
1RM BP (kg)	85.63 ± 12.66	106.25 ± 15.98	78.75 ± 10.90	89.58 ± 9.41	71.82 ± 5.61	74.09 ± 14.11

Notes: BM = body mass; BF% = body fat percentage; BH = body height; CMJ = countermovement jump; HS = handgrip strength; 1RM BP = one repetition maximum bench press.
